# Age-related effects on event-related brain potentials in a congruence/incongruence judgment color-word Stroop task

**DOI:** 10.3389/fnagi.2014.00128

**Published:** 2014-06-17

**Authors:** Montserrat Zurrón, Mónica Lindín, Santiago Galdo-Alvarez, Fernando Díaz

**Affiliations:** Department of Clinical Psychology and Psychobiology, University of Santiago de CompostelaSantiago de Compostela, Spain

**Keywords:** aging, Stroop task, event-related potentials

## Abstract

We examined the event-related brain potentials elicited by color-word stimuli in a Stroop task in which healthy participants (young and old) had to judge whether the meaning and the color of the stimulus were congruent or incongruent. The Stroop effect occurred in both age groups, with longer reaction times in the older group than in the young group for both types of stimuli, but no difference in the number of errors made by either group. Although the N2 and P3b latencies were longer in the older than in the younger group, there were no differences between groups in the latencies of earlier event-related potential components, and therefore the age-related processing slowing is not generalized. The frontal P150 amplitude was larger, and the parietal P3b amplitude was smaller, in the older than in the younger group. Furthermore, the P3b amplitude was maximal at frontal locations in older participants and at parietal locations in young participants. The age-related increase in perceptual resources and the posterior-to-anterior shift in older adults support adaptive reorganization of the neural networks involved in the processing of this Stroop-type task.

## INTRODUCTION

During normal aging, structural and functional changes are produced in the brain and there is a decline in various cognitive processes, including a decline in performance of executive tasks (Raz, 2005; Reuter-Lorenz and Park, 2010; Fabiani, 2012).

The Stroop task, which is a classical test of executive function, involves presentation of stimuli with two dimensions (word meaning and color) that may conflict (incongruent color-word stimuli, e.g., the word red written in blue) or overlap (congruent color-word stimuli, e.g., the word red written in red). Participants are usually asked to respond to the color of the stimulus and ignore the meaning. Consistently, and regardless of age, the response time is longer for incongruent stimuli than for congruent stimuli (Stroop effect).

The Stroop test is sensitive to the cognitive decline associated with normal aging, as demonstrated by the fact that the behavioral response to congruent and to incongruent stimuli is slower, and the Stroop effect is larger in older people than in young people (see MacLeod, 1991; Van der Elst et al., 2006; Peña-Casanova et al., 2009).

The generalized slowing theory has traditionally been used to explain the behavioral decline observed in the Stroop task in elderly people. This theory is mainly based on reaction times (RTs), which provide a measure of the overall cognitive processing speed. In this theory, it is assumed that the slower information processing in older than in young adults is generalized and gradual from the earliest stages of processing (Salthouse, 1996).

However, studies using the event-related potential (ERP) technique have not supported an age-related general slowing. The ERP technique records changes in the brain electrical activity associated with stimulus processing and with the preparation and execution of motor responses, with a high temporal resolution in the order of milliseconds. This enables examination of the time course of the various stages of stimulus processing.

Falkenstein et al. (2006) found that, in choice RT tasks, the longer RT in elderly than in young adults was exclusively associated with delays in the latency of motor ERP components. Moreover, the N2b and P3b (or P300) components, which are recorded during oddball tasks (in which participants are required to identify an infrequent stimulus that differs from the other, frequent stimulus, in some characteristic) and which are related to stimulus evaluation and categorization processes in working memory, showed delayed latencies in older relative to younger subjects. In contrast, the earlier components associated with the most basic perceptual processes did not show differences between the age groups (Amenedo and Díaz, 1998; for a review, see Friedman, 2012).

Studies that have analyzed the ERP components elicited in response to congruent and incongruent word-color stimuli are scarce and provide inconsistent results. Using a color-word Stroop task, Kray et al. (2005) and Eppinger et al. (2007) found that the Ni latency was longer in a group of older participants than in a group of young participants (Ni is a negative wave identified at around 200–600 ms in the incongruent *minus* congruent difference waveforms); in contrast, West and Alain (2000) found no differences between older and younger adults in the P300 latency.

The congruence/incongruence judgment Stroop task, in which the participants have to decide whether both dimensions of the stimulus (color and meaning of the word) were consistent, has also proved to be sensitive to age effects. In a comparison of a group of middle-aged participants (41–61 years) and a group of young participants (21–39 years), Mager et al. (2007) found a longer RT and a larger Strooop effect, as well as longer latencies for the Ni and N450 ERPs components in the older than in the younger group; however, they did not observe any differences between groups in the latencies of P300 and occipital N1. There were also no differences between groups in the amplitudes of occipital N1; in contrast, the P300 amplitude was smaller in middle-aged than in young participants, and in the former, the amplitude was maximal at Fz, although in the younger participants, the amplitude was maximal at Pz. These data on the P300 amplitude are consistent with observations in older participants in the oddball paradigm (Fabiani and Friedman, 1995; Amenedo and Díaz, 1998; for review, see Friedman, 2012), showing a typical topographic pattern: young-parietal/old-frontal.

In studying the speed of processing in the Stroop test, the advantage of the congruence/incongruence judgment Stoop task is that the participants must focus their attention on both dimensions of the stimulus, which reduces the inhibitory component of the classical task (requiring response to the color and ignoring the meaning of the word), while maintaining the conflict between incongruent color words.

In the present study, we measured RT and number of errors, as well as the latency and amplitude of the ERP components elicited in response to congruent and incongruent color-word stimuli during the execution of a congruence/incongruence judgment Stroop task by young (age range: 19–24 years old) and older participants (age range: 60–79 years old). The aim of the study was to determine whether the age-related slowing in the execution of this type of task is associated with a generalized slowing of stimulus processing or if it only affects certain processes. We also tested whether the amplitude and topography of the P300 followed the pattern (Young-parietal/Old-frontal) previously observed in the oddball paradigm, also observed by Mager et al. (2007) in middle-aged participants during execution of a congruent/incongruent judgment Stroop task.

The working hypotheses were as follows: (1) the RT to the congruent and incongruent color-word stimuli will be longer, and the Stroop effect will be greater in magnitude, in older participants than in young participants, indicating that the processing speed is slower in the older group; (2) there will be no differences between older and younger participants for the latencies of early ERP components; (3) the latencies of late ERP components will be longer in elderly than in young adults, showing that the slowed processing of the color-word stimuli in older people is not generalized and only affects certain processes; and (4) the P300 amplitude will be smaller in older than in younger participants, and the distribution will be parietal in the younger participants and frontal in the older participants.

## MATERIALS AND METHODS

### PARTICIPANTS

Eleven elderly adults (three female; mean age = 67.6 years; SD = 6.4) and eleven young adults (three female; mean age = 20.5 years; SD = 1.4), matched in years of education (all university-educated), and with a medium-high socioeconomic status, participated voluntarily in the study. All had normal or corrected to normal vision (but did not use contact lenses). All were healthy, with no history of neurological or psychiatric disorders or drug abuse, and were not receiving medication at the time of participating in the study (nor had received any in the preceding weeks). The participants were also required to abstain from consuming drugs/alcohol/caffeine and nicotine prior to testing, and none reported fatigue caused by lack of sleep. None of the participants were familiar with the protocols used in the study. All participants were self-reported right-handed.

All participants were recruited from the university population (University of Santiago de Compostela): the young participants were undergraduate students and the older participants were attending courses on scientific and/or artistic subjects aimed at older people and imparted by university lecturers.

The 11 young participants in the study were selected in a semi-random manner (to obtain a sample matched in number of participants, sex, education and socioeconomic status with the older adults group) from an initial sample of 21 participants involved in a previous study (Zurrón et al., 2009).

### TASK AND STIMULI

One hundred and four stimuli were presented to the participants in two blocks (each comprising 52 stimuli), with a rest period of 90 s between the blocks. The stimuli were the Spanish words “azul” (blue), “verde” (green), “rojo” (red), and “gris” (gray; each word was displayed 26 times), which were printed in one of these four colors on a black background, so that the stimulus features (word color and word meaning) were either congruent (the color and meaning matched) or incongruent (the word and color did not match). Half of the stimuli were congruent and half incongruent. The stimuli were presented in a random order and were displayed for 200 ms, with a 2000 ms inter-trial interval. The screen was black between the end of one stimulus and the onset of the next.

The color-words were displayed on a flat 19-inch monitor placed one meter from the participant’s face. The stimuli were presented in the center of the screen in upper case letters (Helvetica 2 font); each letter was approximately 2 cm wide and 3.5 cm high and subtended an angle of 1.15 × 2.01 degrees of arc. The participants were asked to press as quickly and accurately as possible, with one hand, a button on a response pad if both dimensions of the stimulus were incongruent, and to press another button, with the other hand, if both dimensions of the stimulus were congruent. Prior to EEG recording, a trial run was carried out with six stimuli to establish whether the participants understood the instructions and responded correctly.

### ELECTROENCEPHALOGRAPHY RECORDING

Prior to the task, each participant was seated in a comfortable chair in an electrically shielded laboratory with attenuated sound and lighting levels. The participants were instructed not to move during the recording session and to look at the center of the screen.

Each participant was fitted with a cap with a nose reference and a frontopolar ground, for recording the EEG activity at 30 electrode sites corresponding to the 10–20 system. Eye movements were recorded simultaneously with additional electrodes placed above and below the left eye (VEOG) and at the outer canthus of each eye (HEOG). All impedances were reduced to 5 KΩ or less in order to obtain good quality recordings, and care was taken to avoid damaging the participant’s scalp.

The EEG signals were passed through a 0.1–30 Hz band-pass filter before being sampled at 500 Hz; the digitalized signal was then stored. Ocular artifacts were corrected off-line with the algorithm of Semlitsch et al. (1986). Segments of 1600 ms (200 ms pre-stimulus) were extracted from the EEG, and the pre-stimulus interval was defined as the baseline.

Signals exceeding ± 100 μV were automatically excluded from the averages, and only the epochs corresponding to the stimuli with correct responses were taken into account. Between 45 and 50 epochs were included in the ERP averages for each type of stimulus (congruent and incongruent), and there were no differences between the numbers of epochs for either type of stimulus or age group.

### DATA ANALYSIS

**FIGURE 1:**
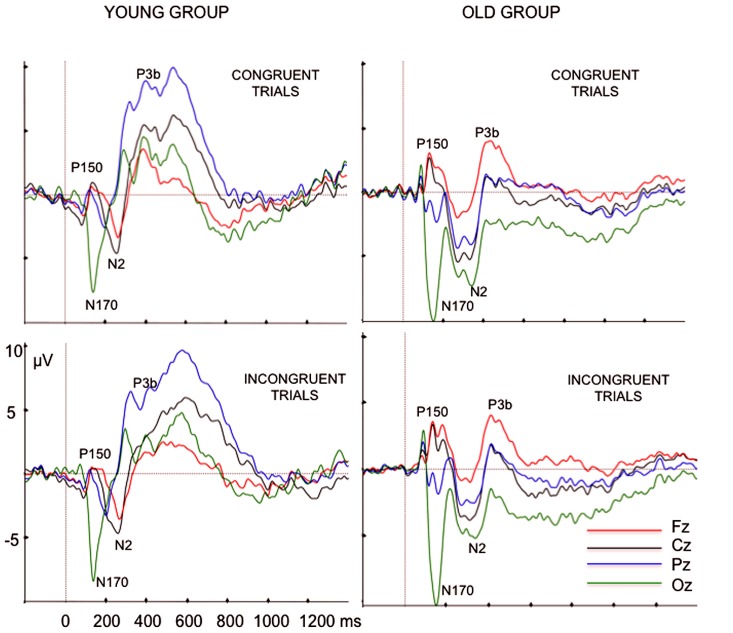
**Grand average ERP waveforms, at Fz, Cz, Pz, and Oz electrode sites, elicited by congruent color words and incongruent color words in the young and old participants**.

After visual inspection of the grand averages, we identified the frontal P150-occipital N170 complex in the 120–180 ms post-stimulus time interval (see Joyce and Rossion, 2005, for a demonstration that both waves comprise a single component). The P150 peak latency and amplitude were measured at Fz, whereas N170 parameters were measured at Oz (see **Figure 1**).

**FIGURE 2:**
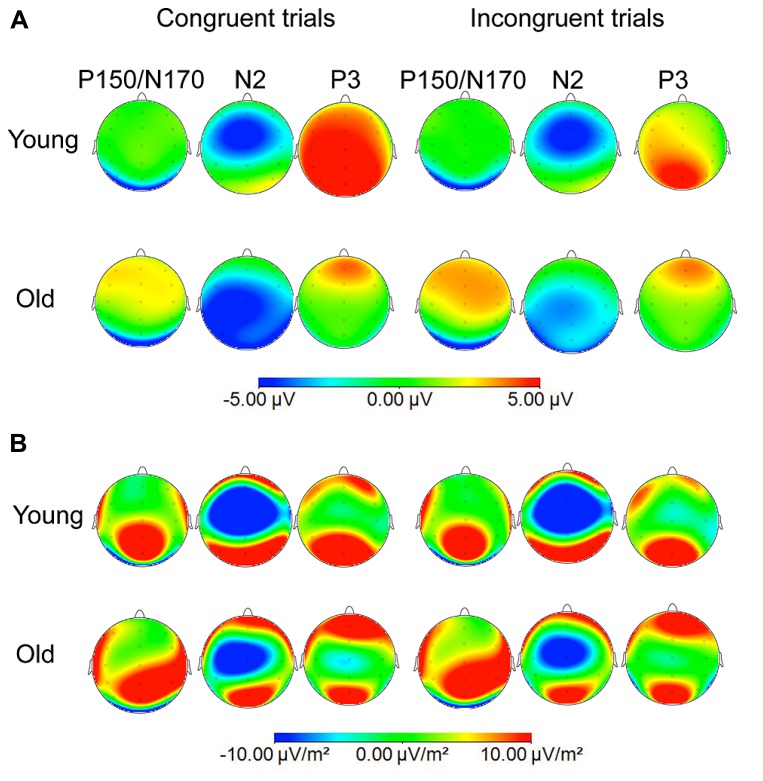
**Voltage (A) and current source density (CSD; B) maps at the maximum peaks of P150/N170 (140 ms for young and old groups), N2 (260 ms for young group and 320 ms for old group), and P3b (390 ms and 440 ms for young and old participants, respectively) to congruent and incongruent stimuli**.

N2 showed a wider scalp distribution in older than in young participants (see **Figure 2**). The N2 peak amplitude was measured at Fz and Cz locations, as this wave was better defined in both age groups at these electrode sites, and the N2 peak latency was measured at Cz electrode site. At Pz and Oz electrodes sites, the N2 component was only identified in the older participants (see **Figure 1**). The N2 component was always identified as a negative voltage wave, which occurred prior to the P3b component, in the 200–350 ms post-stimulus interval. Once it was confirmed that P3b was reliably identified at all the midline electrode sites in the 360–600 ms post-stimulus interval, the P3b peak amplitude was measured at Fz, Cz and Pz, whereas peak latency was only measured at Cz electrode site.

In the ERP waveforms of the young participants (see **Figure 1**), we observed three positive components (between 300 and 800 ms) that had been identified in a previous study (Zurrón et al., 2009): first P3, P3b, and PSW. Of these, only the P3b component was identified at Fz, Cz, and Pz electrode sites. The first P3 component was recorded at Pz and Oz, at between 290 and 330 ms; the PSW was centroparietal with maximal peak amplitude between 540 and 600 ms. In contrast, in the older participants we only observed a positive component at the three midline electrodes, between 300 and 800 ms, (P3b component). Therefore, in the present study, we only evaluated the parameters of the P3b component in the latency range between 300 and 800 ms, as this was the only component identified in both young and older participants.

### STATISTICAL ANALYSES

The RTs, the number of errors, the peak latencies of P150, N170, N2 and P3b, and the amplitude of P150 and N170 were examined by mixed analysis of variances (ANOVAs), with Group as the between-subject factor (two levels: young and old groups), and Congruence as the within-subject factor (two levels: congruent and incongruent).

As N2 and P3b components presented a wider scalp distribution than P150 y N170, N2, and P3b was measured in more than one electrode site, and therefore the statistical analyses included the within-subject factor Electrode. Thus, the amplitudes of N2 and P3b components were analyzed by mixed ANOVAs, with Group as the between-subject factor (two levels: young and old groups) and Congruence (two levels: congruent and incongruent) and Electrode (two levels for N2: Fz and Cz, and three levels for P3b: Fz, Cz, and Pz) as within-subject factors.

Whenever the ANOVAs revealed significant results, pairwise comparison of means (adjusted to Bonferroni correction) was carried out to identify the source of the differences. Greenhouse–Geisser corrections were applied to the degrees of freedom in all cases in which the condition of sphericity was not met. In these cases, the original degrees of freedom were presented together with the corrected p and ∊ values. Differences were considered significant at *p* ≤ 0.05. All statistical analyses were carried out with SPSS (version 19.0).

The RT data and the number of errors committed by two older participants were not stored correctly in the recording session and the data were lost. Some ERP latency and amplitude values were also lost as it was not possible to identify all components in the 22 participants. However, all analyses included data from at least eight participants per group.

## RESULTS

### PERFORMANCE (SEE TABLE 1)

The mean values and standard deviations (SDs) of the RT and the number of errors made by participants in both groups are shown in **Table 1**. It can be noticed that the older group showed longer RTs than the young group, and also longer RTs for incongruent than for congruent stimuli (Stroop effect). Notably, SD was larger for elderly than for young participants in RT, an index of the greater variability of response in the first group.

**Table 1:** Mean values and standard deviations (in brackets) for RT (in ms) and number of errors, in response to congruent (C) and incongruent (IC) stimuli, in old and young participants.

	RT C	RT IC	RT IC vs. RT C (Stroop effect)	Errors C	Errors IC
Young	587 (88)	647 (79)	***60	2.7 (2)	2.3 (2)
Old	827 (134)	951 (170)	***124	2.9 (2)	1.7 (2)
Old vs. Young	**240	***304	*64	–	–

The “Old vs. Young” row provides the difference between age groups in RT and Stroop effect means. The “RT IC vs. RT C” column provides the differences between types of stimuli in RT means (the Stroop effect) for each group and between age groups. Asterisks indicate that differences were statistically significant: *p < 0.05; **p < 0.01; ***p < 0.001.

#### Reaction time

The mixed ANOVA (Group × Congruence) revealed that the Group factor [*F*(1,18) = 28.5, *p* < 0.001], the Congruence factor [*F*(1,18) = 56.5, *p* < 0.001], and the Group × Congruence interaction [*F*(1,18) = 6.67, *p* ≤ 0.019] had significant effects on RT. The RTs were significantly longer in the elderly group than in the young group, and they were also significantly longer for incongruent stimuli than for congruent stimuli. As the Group × Congruence interaction was significant, we investigated whether the magnitude of the Stroop effect was greater in the older participants than in the young participants. For this purpose, we subtracted the RT to the congruent stimuli from the RT to the incongruent stimuli (mean for older participants = 123.7 ms, mean for young participants = 60.4 ms), and we analyzed the differences between groups with a *t*-test for independent samples [*t*(18) = -2.4, *p* ≤ 0.034]: the values of this variable were significantly larger for the older group than for the younger group. Therefore, the magnitude of the Stroop effect was significantly greater in the older participants than in the young participants.

#### Number of errors

No significant effects of the factors or of their interactions were found for the number of errors.

### EVENT-RELATED POTENTIALS (SEE TABLES 2 AND 3, AND FIGURES 1 AND 2)

The mean values (and the SD) of the latencies and amplitudes of the ERP components are shown in **Tables 2** and **3**. The ERP waveforms (**Figure 1**) and the maps of voltages and current densities (at the peak latency of each component; **Figure 2**) are shown for each group and type of stimulus.

**Table 2:** Mean values and standard deviations (in brackets) for P150, N170, N2, and P3b latencies (in ms), to congruent and incongruent stimuli, in old and young participants.

		P150 (Fz)	N170 (Oz)	N2 (Cz)	P3b (Cz)
Young	Congruent	148 (14)	145 (26)	262 (32)	391 (19)
	Incongruent	141 (15)	145 (25)	258 (28)	412 (52)
Old	Congruent	140 (16)	152 (17)	322 (31)	440 (44)
	Incongruent	143 (21)	151 (14)	325 (31)	427 (37)

**Table 3:** Mean values and standard deviations (in brackets) for P150, N170, N2, and P3b amplitudes (in μV), to congruent and incongruent stimuli, in old and young participants.

		P150 (Fz)	N170 (Oz)	N2 (Fz)	N2 (Cz)	P3b (Fz)	P3b (Cz)	P3b (Pz)
Young	Congruent	2.1 (1.6)	-10.9 (5.7)	-4 (3.2)	-6.2 (4.5)	4.3 (2.9)	5.9 (3.7)	9.8 (4.6)
	Incongruent	1.9 (1.3)	-11.1 (6.9)	-4.4 (2.9)	-6.4 (4.7)	3.2 (2.9)	4.3 (3.7)	8.5 (4.4)
Old	Congruent	4.3 (1.7)	-11.6 (7.5)	-2.4 (4.2)	-6.9 (5)	5.3 (3.3)	3.1 (3.4)	2.8 (3.1)
	Incongruent	4.6 (1.3)	-11.5 (7.9)	-1.2 (3)	-4.6 (4.1)	5.8 (2.9)	3.7 (3.1)	3.5 (2.7)

#### Latencies

The mixed ANOVAs (Group × Congruence) did not show any significant effect of the factors or their interaction on P150 and N170 latencies.

The mixed ANOVAs revealed that the Group factor had a significant effect on N2 latency [*F*(1,20) = 25.6, *p* < 0.001] and P3 latency [*F*(1,20) = 5.12, *p* < 0.035], which was significantly longer in older adults than in young adults.

#### Amplitudes

The mixed ANOVA (Group × Congruence) revealed that the Group factor [*F*(1,16) = 15.1, *p* ≤ 0.001] had a significant effect on the P150 amplitude, which was significantly larger in older adults than in young adults.

The mixed ANOVA did not show any significant effect of the factors or of their interaction on N170 amplitude.

In relation to N2 amplitude, the ANOVA (Group × Congruence × Electrode) revealed that Electrode factor [*F*(1,19) = 10.46, *p* ≤ 0.004], and the Group × Congruence interaction [*F*(1,19) = 4.77, *p* ≤ 0.042] were significant. The N2 amplitude was significantly larger at the Cz than at the Fz electrode site, and only in the elderly adults it was significantly smaller to incongruent than to congruent stimuli (*p* ≤ 0.019).

For the P3b amplitude, the ANOVA (Group × Congruence × Electrode) revealed significant effects of the factor Electrode [*F*(2,40) = 4.76, ∊ = 0.7, *p* ≤ 0.025], and the Group × Electrode [*F*(2,40) = 17.49, *p* < 0.001] and Group × Congruence [*F*(1,20) = 4.44, *p* ≤ 0.048] interactions. The mean pairwise comparisons showed that the amplitudes were significantly smaller in response to incongruent than to congruent stimuli only in the young adults (*p* ≤ 0.05); at Pz, the amplitudes were significantly smaller in the older group than in the young group (*p* ≤ 0.001); and in the young adults the amplitude at Pz was significantly larger than at Fz (*p* ≤ 0.001) and Cz (*p* ≤ 0.001), whereas in older adults the amplitude was significantly larger at Fz than at Cz (*p* ≤ 0.031) and at Pz (*p* ≤ 0.05).

## DISCUSSION

### PERFORMANCE

In the Stroop congruence/incongruence judgment task, the RTs to both types of stimuli (congruent and incongruent) were longer, and the magnitude of the Stroop effect was greater in the older than in the younger participants. This is consistent with the findings reported by Mager et al. (2007) for a comparison between young and middle-aged participants, and with the differences between young and elderly adults observed in the classic versions of the Stroop task (see MacLeod, 1991; Van der Elst et al., 2006; Peña-Casanova et al., 2009). The results also support our first working hypothesis (see Introduction).

Hasher and Zacks (1988) reported that a reduced ability to inhibit irrelevant information in older adults explained the poorer performance of the Stroop task by older than by young participants. However, our results do not support this explanation, for the following reasons. First, our congruence/incongruence judgment task removes the inhibitory component for the meaning of the word; consequently, the delayed behavioral response in elderly people relative to young people, in the Stroop-type task, may be due to an age-related decrease in processing speed (Verhaeghen and De Meersman, 1998). Second, there were no differences between the two age groups in the number of errors, which is consistent with data suggesting that the effect of aging is more closely related to the speed of response than to accuracy measures (Van der Elst et al., 2006), perhaps because accuracy prevails over speed in older people.

### ERP LATENCIES

We did not find any differences between older and young participants in the latencies of the early components P150 and N170. However, the latencies of the late components N2 and P3b were longer in the older than in the young participants. These results therefore confirm that the slowed information processing in the Stroop task is not generalized in older people.

The frontal P150-occipital N170 complex has been associated with processing of complex visual stimuli such as words and faces (Schendan et al., 1998; Bentin et al., 1999; Rossion et al., 2003; Joyce and Rossion, 2005; Dien, 2009). Of the two components that form the complex, N170 is the most commonly studied. Thus, N170 in response to words is considered specifically related to sub-lexical perceptual processes (Dien, 2009). As no differences were observed between groups in the P150–N170 complex latencies, the neural processing of word-color stimuli may not be slower in older than in young people, at least until this stage of perceptual processing.

N2 and P3b components are observed in all tasks in which attention must be focused in order to make a decision (Ritter et al., 1979; Luck and Kappenman, 2012), and they have been associated with evaluation and categorization of the stimuli in working memory (Kutas et al., 1977; Hillyard and Kutas, 1983; Folstein and van Petten, 2008).

In the present study, in line with our hypotheses and with the results obtained for some studies using the oddball paradigm (for reviews, see Polich, 1996; Friedman, 2012), the N2 and P3b latencies were longer in older than in young participants. This appears to support the idea that the neural networks underlying cognitive processes of evaluation and categorization of color-word stimuli act more slowly in older than in young participants.

The age-related delays in N2 and P3b latencies (around 60 ms for both components and stimulus conditions) account for only a part of the RT delay in the older group relative to the young group, as the average difference was 264 and 328 ms for congruent and incongruent stimuli, respectively. Consequently, most of the delay in RT might be attributed to the response preparation and execution stages, which is consistent with previous results (Falkenstein et al., 2006).

Future studies should focus on the delay in the RT associated with response processes, by obtaining movement-related potentials, especially considering that the variability in RT is of greater magnitude in older than in young participants. For example, it would be interesting to contrast the effect of motor preparation comparing silent responses tasks (see Amato et al., 2013) with overt responses tasks. In addition, the study of motor ERP components would probably shed light on the causes of the increased magnitude of the Stroop effect obtained in older with respect to young participants.

### ERP AMPLITUDES

With respect to the amplitudes, we observed important differences between young and older participants. The frontal P150 amplitude was larger in the older participants than in the young participants. This may indicate that the older participants dedicated more neural resources to sub-lexical perception of the color-words than the young participants.

The P3b amplitude at Pz was larger in the younger group than in the older group, this result is consistent with previous findings and with our hypothesis. Walhovd et al. (2008) showed that the reduction in P3b amplitude in elderly relative to young participants in oddball tasks was not caused by a latency-jitter effect but that it was a genuine phenomenon of the aging process. These authors attributed the result to a reduction in the neural resources allocated to the process of categorization of the target stimuli with age. In the context of the Stroop task, the P3b amplitude is sensitive to categorization of color words as congruent or incongruent (Zurrón et al., 2009). Thus, smaller P3b amplitudes in response to both types of color-word stimuli in older than in younger participants may indicate that the older participants had fewer resources available to dedicate to the categorization process.

We also observed differences between the two groups of participants with respect to the ERP amplitude in response to congruent and incongruent stimuli. In older participants, the N2 amplitude was smaller to incongruent stimuli than to congruent stimuli. In young participants, the amplitude of P3b was smaller to incongruent stimuli than to congruent stimuli, in accordance with previous findings (see Zurrón et al., 2009). Unlike the young participants, older participants did not show any differences in P3b amplitude between incongruent and congruent stimuli, perhaps because of a possible ceiling effect, given the small magnitude of P3b in older participants. The smaller amplitudes to incongruent than to congruent stimuli may be interpreted in terms of greater difficulty in evaluating and classifying the incongruent stimuli, as the amplitude of P3b decreases with the difficulty of the task (Donchin, 1981; Johnson, 1986; Donchin and Coles, 1988; Verleger, 1988).

Moreover, the topographical distribution of P3b was different in the two groups, thus confirming our hypothesis: in the older participants, the P3b amplitude was maximal at frontal locations, whereas in young participants it was maximal at parietal locations. These differences between groups can be seen in the ERP waveforms, as well as in the voltage and current density maps.

The more frontal topographical distribution of the P3b with age, the reduced P3b amplitude in older than in young adults in parietal but not in frontal electrode sites, and the larger amplitude of P150 at Fz in the older than in the young participants may reflect overactivation of frontal areas, which is consistent with the posterior–anterior shift in aging (PASA) hypothesis (Grady et al., 1994; Davis et al., 2008). The PASA hypothesis is included within the scaffolding theory of aging and cognition (STAC; see Park and Reuter-Lorenz, 2009), which suggests that the neural networks that sustain cognitive processes undergo modifications to adapt to the changes produced in the brains of older participants and to maintain functions as well as possible, although the new scaffolded networks may be less efficient than the original ones.

In summary, the RT data indicate that older participants processed information more slowly than younger participants in a Stroop congruence/incongruence judgment task. Psychophysiological data obtained with the ERP technique indicate that the age-related slowing is not generalized, since it does not affect the neural networks underlying perceptual processes, but that it does affect the neural networks underlying evaluation and categorization cognitive processes. Moreover, the ERP data confirm changes in the neural functioning of the older participants during the processing of a congruence/incongruence judgment color-word Stroop task. As the number of errors was similar in both age groups, such neurofunctional changes might be explained by a compensatory reorganization of brain neural networks with aging, consistent with the neural scaffolding hypothesis.

## Conflict of Interest Statement

The authors declare that the research was conducted in the absence of any commercial or financial relationships that could be construed as a potential conflict of interest.
